# QIP on Improving Access to Physical Health Pathways, Services, and Resources for Inpatients in KMPT

**DOI:** 10.1192/bjo.2025.10418

**Published:** 2025-06-20

**Authors:** Olubunmi Olure, Maham Zahid, Hussein Al-Siraj, Tonye Ajiteru, Chiara Rubino

**Affiliations:** KMPT, Kent, United Kingdom

## Abstract

**Aims:** Mental health patients are often severely unwell presenting with significant risks to their health and multi-morbidity/complex health needs. Currently, at KMPT there is a lack of access to the right care at the right time with a lot of silo mentality and fragmented services. Feedback from junior doctors includes:

1. Lack of clarity or easy accessibility of relevant physical health-related trust policies.

2. Unclear referral pathways between KMPT and acute trust relevant physical health specialties.

A survey was conducted amongst resident doctors in KMPT to identify the scale of the problem. We aim to improve:

Knowledge and awareness of access to physical health interventions on KMPT inpatient wards.

Systems and protocols for liaison and consultation with physical health care teams/specialists.

Patient safety and patient experience.

**Methods:** Based on feedback, we felt that the best solution to increase staff confidence in managing physical health problems and increase their awareness of what is available, would be to create an online easy to access page where everything is centralized in one place. In turn this would allow for a better patient experience and improved patient safety on our wards.

The creation of a staffroom page for all physical health resources for KMPT staff. As part of our staffroom page, there will be live links to relevant policies, IT applications, IT systems and relevant referral pathways contact information

**Results:** Liaise with the Trust digital team to create a folder on the intranet for resident doctors containing all the collated resources. This includes:

Collated contacts for referral pathways for the 5 Acute trusts in Kent.

Collected relevant pathology and imaging request forms and made an electronic copy for easy access.

Collated all the trust policy guidelines relating to physical health and put in a folder for easy access for resident doctors.

Liaised with the Trust physical health and infection control team for one click easy access on the Trust.

We have presented to the Trust Clinical director and Head of Psychiatry, as well as Urgent care Community of practice for necessary support for the project.

**Conclusion:** We would go live on the trust intranet next month and then roll out the second PDSA to resident doctors.

